# Determination of Total Soluble Sugars in Pteridophytes Using the Anthrone Method

**DOI:** 10.1002/cpz1.70373

**Published:** 2026-04-22

**Authors:** Pablo Denova‐Lozano, Alejandra Chamorro‐Flores, Amparo B. Cerón‐Carpio, Analilia Arroyo‐Becerra

**Affiliations:** ^1^ Benemérita Universidad Autónoma de Puebla Facultad de Ciencias Biológicas México; ^2^ Laboratorio de Genómica Funcional y Biotecnología de Plantas, Centro de Investigación en Biotecnología Aplicada, Instituto Politécnico Nacional Ex‐Hacienda San Juan Molino Carretera Estatal Km 1.5, Santa Inés‐Tecuexcomac‐Tepetitla Tlaxcala México; ^3^ Benemérita Universidad Autónoma de Puebla, Herbario y Jardín Botánico Vicerrectoría de Investigación y Estudios de Posgrado México

**Keywords:** anthrone method, pteridophytes, soluble sugars, stress tolerance

## Abstract

Sugars serve as crucial integrators of both internal and environmental signals in plants, shaping the regulation of diverse physiological processes that occur throughout the plant's life span, from early embryogenesis to later senescence stages. During evolution, plants have developed various strategies to cope with abiotic stress. For example, the accumulation of osmolytes such as soluble sugars, which help protect against oxidative stress, stabilizes cellular membranes and preserves enzymes in the dry state. Precise quantification of total sugars is therefore essential for elucidating the biochemical and physiological strategies of plants in response to different conditions. Here, we present a detailed protocol for extracting and quantifying total soluble sugars in pteridophytes (lycophytes and ferns) from small amounts of leaf tissue (10 mg fresh tissue) using the anthrone method, a colorimetric assay in which carbohydrates react with the anthrone reagent under acidic conditions to form a blue‐green complex whose concentration is measurable by spectrophotometry. The procedure was adapted for small sample volumes, incorporating ethanol extraction, preparation of a glucose standard curve, and absorbance measurement at 620 nm in 96‐well plates. Quantification of total sugars in pteridophytes is essential for understanding the changes in metabolic responses. Likewise, using small amounts of plant tissue optimizes sugar extraction in plants with low biomass and minimizes impact on plant populations. © 2026 The Author(s). *Current Protocols* published by Wiley Periodicals LLC.

**Basic Protocol 1**: Extraction of fresh plant tissue

**Basic Protocol 2**: Reaction with anthrone and measurement

**Support Protocol 1**: Preparation of anthrone reagent

**Support Protocol 2**: Preparation of the glucose standard curve

**Basic Protocol 3**: Calculation of total sugar concentration

## INTRODUCTION

Pteridophytes (lycophytes and ferns) are the second‐largest group of vascular plants worldwide, with ∼13,000 species (Qian et al., 2021). This group of plants has developed a series of key innovations and adaptive strategies to cope with abiotic stress (Chen, 2022; Kessler & Siorak, 2007). In plants, including pteridophytes, sugars can serve as energy sources, are transported to sink organs, and act as key components in the coordination of internal and external signals, contributing to the regulation of numerous biological processes throughout the plant life cycle, from embryonic development to senescence (Bolouri‐Moghaddam et al., 2010; Iturriaga et al., 2006; Li & Sheen, 2016; Saddhe et al., 2021; Zhang et al., 2016). Additionally, several studies have demonstrated that sugars have fundamental roles in plants’ responses to stress factors (Jeandet et al., 2022). An increase in sugar concentrations can be considered an indicator of plant preparedness for stress conditions, as they act as osmolytes contributing to radical scavenging, osmotic adjustment, preservation of subcellular structures through cytoplasmic vitrification, carbon storage, stabilization of protein structures, and protection of cellular membranes from damage caused by water loss (Ghosh et al., 2021; Keunen et al., 2013; Oliver et al., 2020; Schwab & Heber, 1984; van den Ende & Valluru, 2008).

For quantification of total soluble sugars, one of the most widely used techniques is the anthrone method, a colorimetric assay based on the reaction of carbohydrates with the anthrone reagent in an acidic medium, which produces a blue‐green complex whose absorbance can be measured spectrophotometrically (Yemm & Willis, 1954). Despite the importance of quantifying total sugar in pteridophytes, few studies have applied the anthrone method to this plant group (Schwab & Heber, 1984; Schwab & Gaff, 1986). Moreover, existing protocols often require large amounts of tissue (e.g., 100 mg of the fern *Ceterach officinarum* Lam et DC.; Schwab & Gaff, 1986), usually do not specify the exact quantity needed, and lack clear methodological descriptions, limiting their reproducibility. Therefore, it is essential to develop a standardized, optimized protocol that enables the reliable determination of total carbohydrates in pteridophytes and other small specific plant tissues and organs using minimal amounts of tissue and a well‐defined methodology.

This protocol aims to determine total soluble sugar content in pteridophytes using the anthrone method, thereby contributing to the understanding of their biochemical and physiological adaptation strategies.

## Basic Protocol 1 EXTRACTION OF FRESH PLANT TISSUE

The extraction of plant tissue is a fundamental step in numerous biochemical and physiological studies, as it enables the isolation of soluble plant compounds for subsequent analysis. This protocol provides a method for extracting sugars from the tissues of lycophytes (*Selaginella pallescens* [C. Presl] Spring, *Selaginella sartorii* Hieron) and ferns (*Pleopeltis madrensis* [J. Sm.] A. R. Sm. & Tejero, *Pleopeltis polylepis* var. *polylepis* [Roem. ex Kunze] T. Moore) using 80% ethanol. The technique was optimized for small sample quantities, making it ideal for laboratory work with limited plant material availability. For this procedure, three biological samples of each species were used, consisting of fresh tissue sections taken from the middle portion of the pteridophytes’ leaves, ensuring equal sample weights across replicates and enabling reliable comparisons.

### Materials

Fresh pteridophyte tissue
80% (v/v) ethanol

Pestle
Refrigerated centrifuge (5415R, Eppendorf, Germany)
1.5‐ml microcentrifuge (Eppendorf) tubes
Vortex mixer

1 Weigh out 10‐mg samples of fresh tissue, place in microcentrifuge tubes, and add 0.5 ml of 80% ethanol to each tube.
  The procedure can be scaled up to grams of tissue while maintaining the proportion of plant material and solutions.
2 Grind up the tissue immediately with a pestle for each sample and centrifuge 10 min at 14,000 rpm, 4°C.
3 Recover the supernatant into a fresh set of microcentrifuge tubes and allow the ethanol to evaporate in open air at room temperature over ∼48 h.
  Centrifuge again to remove any remaining solid residues, if necessary.
  The evaporation time of the ethanol depends on the volume of the extraction used, and vacuum can be used to reduce evaporation time.
4 Resuspend the concentrated residue in 0.5 ml sterile distilled water.

## Basic Protocol 2 REACTION WITH ANTHRONE AND MEASUREMENT

Quantification of sugar content by the anthrone method is a widely used procedure in biochemical studies to determine total sugars in plant extracts. This colorimetric assay quantifies carbohydrates by forming a blue‐green complex, the intensity of which is proportional to the sample's sugar concentration. In the present protocol, the anthrone reagent in an acidic medium is used to react with previously obtained plant tissue extracts (see Basic Protocol 1) and with a standard glucose curve, allowing quantification by comparison. The procedure has been scaled down to small volumes for application in 96‐well microplates, enabling efficient absorbance measurement at 620 nm using a plate reader. For this analysis, the samples were carefully cooled and heated to ensure proper formation of the colored complex, and uniform conditions across replicates were maintained to obtain reliable and reproducible data.

### Materials

Anthrone reagent (see Support Protocol 1)
Sample extract (see Basic Protocol 1)
Glucose standard curve (see Support Protocol 2)

Micropipet
Refrigerator or ice bucket
Vortex mixer (Vortex Mixer Labnet)
Microcentrifuge (Eppendorf) tubes
Thermoblock (DLAB HB150‐52) for microcentrifuge tubes
96‐well microplate
Microplate reader (Thermo Scientific Multiskan GO)

1 Add 1 part of the sample extract of interest to 6 parts anthrone solution.
  In our case, due to the amounts of tissue and samples, the quantities were scaled to 120 µl anthrone solution and 20 µl sample extract.
  Upon addition of the sample to the anthrone, two layers will form.
2 Place the samples at 4°C for 5 min (in a refrigerator or on ice).
3 Remove the samples from the refrigerator or ice and vortex briefly to combine the two layers.
4 Place the microcentrifuge tubes with the samples inside the thermoblock for 10 min at 100°C in a water bath.
  It is important not to fully seal the tubes with their caps. Glass beads can be placed in the tubes to prevent them from becoming completely airtight.
  As the minutes pass, the samples will turn blueish. The color intensity is related to the sample's sugar concentration.
5 After the 10 min are over, rapidly cool the samples on ice.
6 Once the samples are completely chilled, take 140 µl of each sample, including the glucose standard curve samples, and place it in an individual microplate well.
  Care must be taken with the order in which the microplate is filled to avoid confusion.
7 Once all samples have been added to the plate, measure the absorbance at 620 nm.

## Support Protocol 1 PREPARATION OF ANTHRONE REAGENT

The anthrone reagent is an essential component of colorimetric methods for carbohydrate quantification, as it forms a colored complex whose intensity is proportional to the sugar concentration present in the sample. Its preparation requires specific precautions due to the use of concentrated sulfuric acid and the reagent's sensitivity to light. This protocol describes the controlled dilution of sulfuric acid followed by the dissolution of anthrone to obtain the working reagent. As the reagent's stability decreases over time, it is recommended to prepare it immediately before use and store it under appropriate conditions to ensure reproducible and accurate results.

### Materials

Concentrated (98%) sulfuric acid (H_2_SO_4_; CAS no. 7664‐93‐9; Meyer, Mexico)
Sterile distilled water
Anthrone (C_14_H_10_O; CAS no. 90‐44‐8; Sigma‐Aldrich, Germany)

Fume hood
Glass container
Analytical balance

1 Dilute 25 ml of concentrated (98%) H_2_SO_4_ in 10 ml sterile distilled water in a glass container.
  The quantities can be adjusted proportionally to the volume to be used; the volumes depend on the number of samples and their respective weights.
*CAUTION*: The mixing process generates an exothermic reaction and must be performed in a suitable fume hood with efficient ventilation; safety glasses and reagent‐impermeable protective gloves should be worn; and the mixture must be allowed to cool completely in the fume hood before use.
2 Weigh out 0.07 g of anthrone and dissolve it in 35 ml of the diluted sulfuric acid.
  The amount of anthrone can be scaled for the number of samples to be evaluated. Upon adding anthrone to the acid, the reagent turns yellow‐green.
3 Store in a cool place, protected from light, and use as soon as possible.
  The anthrone reagent loses effectiveness over time, so it is advisable to use it as soon as possible to obtain reliable results.

## Support Protocol 2 PREPARATION OF THE GLUCOSE STANDARD CURVE

Preparation of a glucose standard curve is a fundamental step in colorimetric assays aimed at quantifying total soluble sugars in biological samples. This curve establishes a direct relationship between known glucose concentrations and the corresponding absorbance values, enabling accurate estimation of sugar content in unknown plant extracts or other solutions. This protocol outlines the preparation of a glucose stock solution and the execution of a series of controlled dilutions, using sterile materials and careful pipetting techniques to ensure precision and reproducibility of the results.

### Materials

Anhydrous glucose (dextrose; certified ACS; cat. no. D16‐3, Fisher Chemical, Fair Lawn, NJ)
Sterile distilled water

Analytical balance
Microcentrifuge (Eppendorf) tubes
Micropipet

1 Weigh out 5 mg glucose and dissolve it in 50 ml sterile distilled water (to make a 100 µg/ml stock).
2 Prepare a series of dilutions in microcentrifuge tubes to obtain different concentrations, as shown in Table 1.
  When preparing the different dilutions, to obtain a high‐quality standard curve, it is advisable to use new tubes and pipet carefully.

**Table 1:** Glucose Concentrations for the Preparation of the Standard Curve

Tube	Concentration (µg/ml)	Volume of glucose solution (µl)	Volume of distilled water (ml)
Blank	0	0	1
1	0.05	5	0.995
2	1	10	0.99
3	5	50	0.95
4	10	100	0.9
5	20	200	0.8
6	30	300	0.7
7	40	400	0.6
8	50	500	0.5
9	60	600	0.4
10	70	700	0.3
11	80	800	0.2
12	90	900	0.1
13	100	1000	0

## Basic Protocol 3 CALCULATION OF TOTAL SUGAR CONCENTRATION

The calculation of total sugar concentration in biological samples is based on comparing their absorbance values to a previously generated standard curve. This curve, obtained from known glucose concentrations, establishes a linear relationship between absorbance and sugar concentration. This protocol describes the procedure for plotting this relationship and fitting a linear regression line, which can then be used to determine the unknown concentrations in the samples analyzed. The analysis can be performed with specialized software such as CurveExpert, enabling more precise and efficient interpretation of the resulting data.

**Figure 1:**
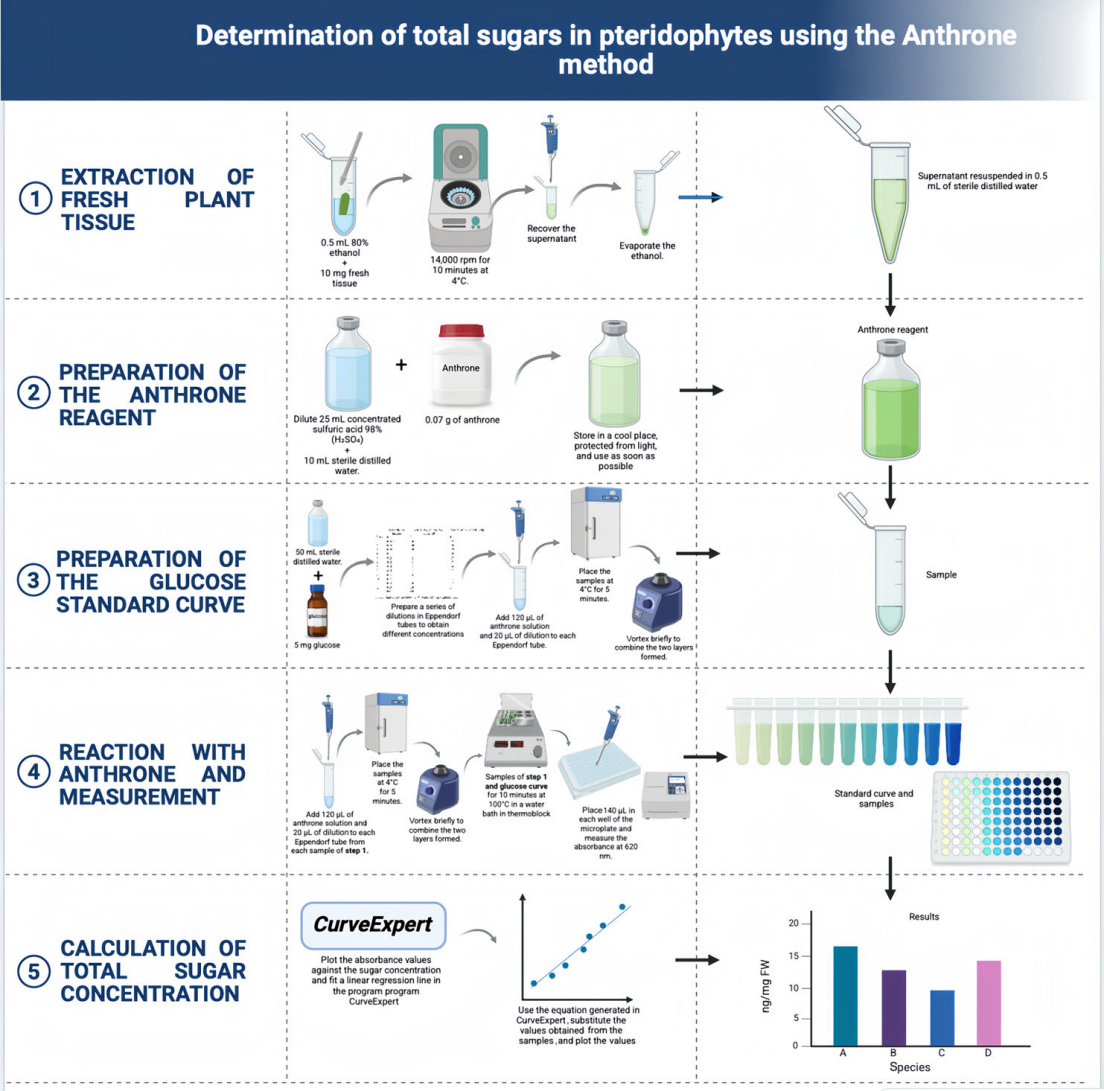
Diagram illustrating the steps for quantifying total sugars by the anthrone method in samples from small tissue samples pteridophyte plants.

**Figure 2:**
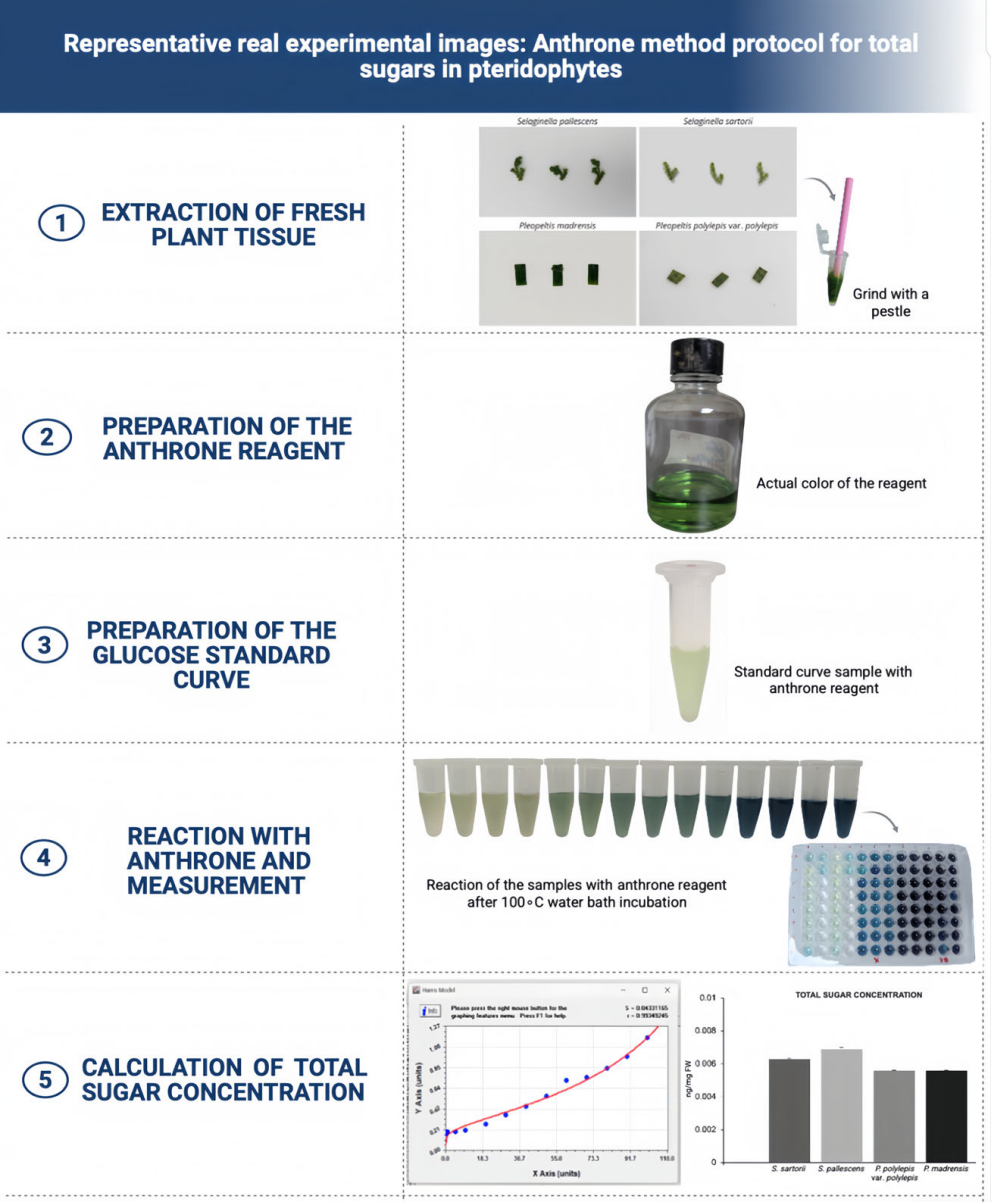
Overview of anthrone method protocol. (1) Images of pteridophytes used to quantify total sugars by the anthrone method. The lycophytes *Selaginella pallescens* and *Selaginella sartorii* and the ferns *Pleopeltis madrensis* and *P. polylepis* var. *polylepis* are shown. (2) The mixture formed with anthrone and diluted sulfuric acid. (3) Sample with anthrone reagent. (4) Color in the samples of standard curve after they have reacted with anthrone. (5) Results obtained through CurveExpert and graphical representation of mean from three biological samples for each species ± standard deviation (SD).

1 Plot the absorbance values against the sugar concentration and fit a linear regression line. A program such as CurveExpert can also be used to obtain the equation for determining sample sugar concentrations.
2 Use the equation generated in CurveExpert and substitute the absorbance values obtained from the samples.
3 Plot the obtained values
  The complete procedure is outlined in Figure 1, and representative experimental images and data obtained for the pteridophytes we investigated are shown in Figure 2 and Table 2.

**Table 2:** Sample Soluble Total Sugar Concentration Data from Lycophytes and Ferns^a^

Species	OD	Concentration	ng/mg FW	Mean	SD
*Selaginella sartorii*	0.196	1.27E‐01	0.006334488	0.006296167	3.98824E‐05
	0.186	1.26E‐01	0.006299125		
	0.174	1.25E‐01	0.006254888		
*Selaginella pallescens*	0.378	1.37E‐01	0.006830638	0.006885578	0.000104952
	0.373	1.36E‐01	0.006819501		
	0.463	1.40E‐01	0.007006595		
*Pleopeltis polylepis* var. *polylepis*	0.055	1.12E‐01	0.005609602	0.005602282	9.16978E‐06
	0.0545	1.12E‐01	0.005605246		
	0.053	1.12E‐01	0.005591996		
*P. madrensis*	0.0554	1.12E‐01	0.005613066	0.005609598	3.00394E‐06
	0.0548	1.12E‐01	0.005607863		
	0.0548	1.12E‐01	0.005607863		

^*a*^ Soluble total sugar concentrations from 10 mg of the lycophytes *S. pallescens* and *S. sartorii* and the ferns *P. madrensis* and *P. polylepis* var. *polylepis*, generated by CurveExpert according to the Harris model: *y* = 1/(*a* + *bx^c^*); coefficient data: *a* = 17.58459974, *b* = –11.1768188742, *c* = 0.087512012.

## COMMENTARY

### Background Information

Having a clear, optimized methodology for small quantities of plant tissue provides a high‐performance alternative, reduces reagent costs, and is even applicable for screening. Importantly, it facilitates the study of organ‐specific physiological processes across different plant groups (such as buds or fine roots) and in plants or tissues with very slow growth or development, in plants or tissues cultured *in vitro*, in tiny plants such as bryophytes, or in minimal samples, thus expanding the scope of research.

### Author Contributions

**Pablo Denova‐Lozano**: Methodology; writing—original draft; formal analysis; visualization. **Alejandra Chamorro‐Flores**: Methodology; validation; visualization; writing—review and editing; software; formal analysis. **Amparo B. Cerón‐Carpio**: Investigation; writing—review and editing; supervision; resources. **Analilia Arroyo‐Becerra**: Conceptualization; investigation; funding acquisition; writing—review and editing; project administration; supervision; resources.

### Conflict of Interest

The authors declare no conflict of interest.

## Data Availability

The data that support the findings of this study are available on request from the corresponding author.
